# Investigating mental health disparities in rural sexual and gender minority adults: protocol for the rural exploration and approaches to LGBTQ + Mental Health (REALM) prospective cohort study

**DOI:** 10.1186/s12889-024-21151-y

**Published:** 2025-02-06

**Authors:** Shoshanna L. Fine, Kalai Willis, Iaah L. Lucas, Kirsten F. Siebach, Jennifer L. Glick, Mariah Valentine-Graves, Savannah Winter, Michael Smith, Thomas Waltz, Gina Bailey Herring, Marissa Hannah, Holly C. Wilcox, Travis Sanchez, Stefan D. Baral, Sarah M. Murray

**Affiliations:** 1 Department of Mental Health, Johns Hopkins Bloomberg School of Public Health, Baltimore, MD USA; 2 Department of Epidemiology, Johns Hopkins Bloomberg School of Public Health, Baltimore, MD USA; 3 Programs, Research, & Innovation in Sexual Minority (PRISM) Health, Rollins School of Public Health, Emory University, Atlanta, GA USA; 4 Department of Epidemiology, Rollins School of Public Health, Emory University, Atlanta, GA USA; 5 Department of International Health, Johns Hopkins Bloomberg School of Public Health, Baltimore, MD USA; 6 Department of Community Health Science & Policy (CHSP), School of Public Health, Louisiana State University Health Science Center, New Orleans, LA USA

**Keywords:** Mental health, Stigma, LGBTQ, Sexual minority, Gender minority, Rural, Cohort

## Abstract

**Background:**

Sexual and gender minority (SGM) persons experience substantial mental health disparities throughout the life course, including increased vulnerability to depression and suicide. Few existing studies, however, have explored how pervasive experiences of SGM-related stigma, discrimination, and trauma (i.e., minority stress) contribute to adverse mental health outcomes among diverse sub-populations of SGM adults living in rural areas of the United States. This paper describes the protocol for a prospective cohort study, “Rural Exploration and Approaches for LGBTQ + Mental Health (REALM),” that will explore minority stress in relation to mental health conditions and suicidal behaviors among rural SGM adults.

**Methods:**

Online processes will be used to recruit and enroll a diverse sample of up to 2,500 SGM adults aged 18 + living in rural counties and small metropolitan areas in the United States to complete an online baseline survey. This will include: up to 1,000 cisgender sexual minority persons (up to *n* = 500 each cisgender women and cisgender men); and up to 1,500 gender minority persons (up to *n* = 500 persons who were assigned male at birth and identify as a woman, female, and/or transfeminine; up to *n* = 500 persons who were assigned female at birth and identify as a man, male, and/or transmasculine; and up to *n* = 500 persons who identify as some other gender, including non-binary, gender non-conforming, and/or agender regardless of sex assigned at birth). All enrolled participants will subsequently be followed over a 12-month period, with repeated surveys at three-month intervals. Included survey measures will focus on sociodemographic information, mental health, substance use, suicidal behaviors, minority stressors, psychological processes, and other related risk and protective factors.

**Discussion:**

This study presents a critical opportunity to better understand how minority stress contributes to adverse mental health outcomes among populations that remain underrepresented in research and programs in 2024. Results will be used to create more targeted, acceptable, and impactful intervention content and strategies that mitigate stigma, promote mental health, and prevent suicidal behaviors among rural SGM adults.

## Background

Sexual and gender minority (SGM) persons – including but not limited to those who identify as lesbian, gay, bisexual, transgender, and queer (LGBTQ+) – experience a high burden of mental disorders, substance use disorders, and suicidal behaviors [1–3]. Further, a large body of research shows substantial disparities in mental health across the life course between SGM persons and their heterosexual, cisgender peers [1–9]. For instance, the 2022 National Survey on Drug Use and Health noted that in the past year, nearly half of sexual minority adults had a mental disorder compared to one fifth of heterosexual adults, and around one in five had a substance use disorder compared to less than one in ten heterosexual adults [5]. Both mental disorders and substance use disorders have been found to be associated with suicidal behaviors among sexual minority persons [10, 11], and one large comparative study of suicide decedents in the United States (US) suggests that these associations may be stronger among sexual minority persons compared to their heterosexual peers [10]. Similar patterns have been documented among gender minority persons [2, 3, 7, 9]. For example, a recent study included a nationally representative sample of US adults and found that when compared to their cisgender peers, transgender adults reported significantly higher odds of serious psychological distress (aOR = 3.1), recent suicidal ideation (aOR = 5.1), lifetime suicidal ideation (aOR = 6.7), and lifetime suicide attempts (aOR = 4.4) [9].

While the suboptimal mental health and suicidal behaviors experienced by SGM persons are well-documented, the upstream determinants of these disparities have not been extensively studied [12]. The Minority Stress Model suggests that societal marginalization of SGM persons results in specific distal stressors (i.e., external stressors such as discrimination, violence, and harassment) and proximal stressors (i.e., internal stressors such as internalized homophobia or transphobia, anticipated rejection, and identity concealment) which may be responsible for driving the relatively higher prevalence of poor mental health outcomes compared to the general population [13–15]. These stressors are reflective of stigma, a complex social process in which an individual is labeled based on a specific characteristic, linked to a negative stereotype, and set apart from others based on perceived differences, resulting in experiences of discrimination, marginalization, and devaluation [16]. Studies informed by this theory have generated broad evidence for the role of minority stress processes in creating and sustaining disparities in mental health for SGM persons [17–19]. For instance, a systematic review of 30 studies found that discrimination was consistently associated with both greater suicidal ideation and attempts for transgender adults, although the cross-sectional design of most studies precluded causal interpretations [20].

Combining minority stress models with suicide theory can help elucidate modifiable mechanisms through which stigma produces mental health disparities among SGM persons [21, 22]. A multitude of theories for understanding suicide-related outcomes exist, including several “ideation-to-action” theories that conceptualize suicidal ideation and attempts as having distinct underlying processes [23]. The Interpersonal-Psychological Theory of Suicidal Behavior (IPTS), for example, posits that thwarted belongingness and perceived burdensomeness lead to suicidal ideation, while the capability to act precipitates suicide attempts [24]. Importantly, it has been argued that painful and traumatic experiences may desensitize individuals to the fear of pain and death that serves as a deterrent to attempted suicide [24]. This may be particularly relevant for SGM persons given a higher documented prevalence of interpersonal traumas across the life course, including abuse during childhood, bullying victimization, and sexual assault [25–28]. Indeed, IPTS and related theories have increasingly been integrated to guide mental health research among SGM persons. For instance, among gender minority participants in the US Trans Health Survey, Testa et al. found that while distal stressors acted through proximal stressors in relation to suicidal ideation, thwarted belongingness and perceived burdensomeness mediated this relationship [29]. This has led other SGM researchers to conclude that minority stress models alone are insufficient for characterizing the processes that shape SGM suicide disparities [21].

Despite progress, significant gaps remain in understanding mental health disparities among SGM populations; chief among them is the lack of data on SGM persons living in rural areas. This is a critical limitation, as evidence drawn from the general population suggests that suicide rates are higher in rural compared to urban settings, potentially due to increased social isolation, greater access to lethal means, heightened stigma around mental health, and poor access to mental health services [30]. Importantly, the limited studies that do focus on rural SGM populations indicate that mental health disparities may be intensified in these areas: both when compared to rural cisgender, heterosexual adults [31, 32] and when compared to urban SGM populations [33–37]. There are several potential explanations for these patterns. Rural SGM populations may contend with substantial stigma and discrimination, limited social capital, and lower healthcare access and utilization [38–40]. There may also be important differences in mental health and suicide risk factors between SGM and non-SGM persons living in rural areas. For instance, sexual orientation change efforts (e.g., conversation therapy), 80% of which are implemented by religious leaders, have been associated with increased suicidal ideation and behaviors among sexual minority persons [41], and both religiosity and religious services attendance have been found to be higher in rural vs. urban areas [42–44]. While there is evidence that religiosity may be protective against suicide in the broader population [45, 46], it has been found to be associated with greater recent suicidal ideation and attempts for sexual minority young adults [47].

In addition, while existing studies have largely focused on adolescents and young adults [2], most suicide deaths occur among those who are middle-aged [48], mirroring trends in the broader population [49]. Longitudinal research is also limited and again has focused predominantly on adolescents and young adults [50]. Experiences influencing mental health and suicide risk may change across the life course, and cohort effects have been observed among SGM populations. For example, in a national sample of sexual minority persons, those born in recent years (1990–97) were coming out earlier in life, perhaps reflecting broader societal shifts in acceptance [51]. However, this younger cohort did not report fewer distal stressors (e.g., discrimination) or proximal stressors (e.g., internalized stigma) compared to older cohorts (born between 1956–63 and 1974–81), and they also reported a greater prevalence of psychological distress and suicidal behaviors. Assessing a large sample of SGM adults across a wide age range, and following them longitudinally, is critical to understanding variation across the life course in terms of the development and progression of mental health conditions and suicidal behaviors.

Lastly, few studies have adopted an intersectional approach to understanding mental health disparities among SGM persons. The existing literature in this area has predominantly focused on white, cisgender samples, overlooking the unique stressors faced by individuals who experience multiple forces of marginalization [52]. Emerging research, however, has emphasized the ways in which intersecting forces of oppression and marginalization may shape mental health disparities among diverse SGM subpopulations in the US [53–55]. For instance, one longitudinal study conducted among cisgender Black, Latino, and multiracial sexual minority men found that the interaction between racial discrimination and internalized sexual orientation-related stigma contributed to greater levels of depression, anxiety, and alcohol use over the 12-month follow-up period [54]. There is also evidence that minority stress reflected in the experiences of individuals who vary in gender identity, gender expression, and sexual orientation may contribute to further mental health disparities among SGM subgroups. Indeed, several studies have suggested that individuals who identify as both sexual *and* gender minorities (e.g., gay transmasculine persons, bisexual gender non-conforming persons) have significantly worse mental health outcomes compared to those who identify only as sexual *or* gender minorities [56, 57].

In summary, there is a need for longitudinal investigations that characterize the complex and nuanced interrelationships between minority stressors, mental health conditions, and suicidal behaviors over time among heterogeneous rural SGM adults. This is necessary to create more targeted, acceptable, and impactful intervention content and strategies that mitigate stigma, promote mental health, and prevent suicidal behaviors. In response to these needs, the current study will enroll and follow a large and diverse group of SGM adults living in rural and small metropolitan areas in the US. The study has two specific aims: (1) determine whether classes of stigma, discrimination, and traumatic experiences vary across subgroups of rural SGM adults and whether these exposure classes are associated with an increased *prevalence* of depression, suicidal ideation, and suicide attempts; and (2) determine whether the classes identified in Aim 1 are associated with an increased *incidence* of depression, suicidal ideation, and suicide attempts among rural SGM adults.

## Methods/design

### Study design

This prospective longitudinal cohort study, “Rural Exploration and Approaches for LGBTQ + Mental Health (REALM),” will explore minority stress in relation to mental health conditions and suicidal behaviors over a 12-month period among rural SGM adults. We will use online processes to recruit and enroll a diverse sample of up to 2,500 SGM adults living in rural counties and small metropolitan areas in the US to complete an online baseline survey. All enrolled participants will subsequently be followed over a 12-month period, with repeated surveys at three-month intervals. The study has been approved by the Johns Hopkins Bloomberg School of Public Health Institutional Review Board (IRB No. 00022255).

This study builds upon prior collaborations between investigators at the Johns Hopkins Bloomberg School of Public Health and the Programs, Research, and Innovation in Sexual Minority (PRISM) Health team at Emory University’s School of Public Health (e.g., [58–62]). Our team of faculty, staff, and students work collaboratively across institutions, drawing upon research, practice, and lived experience in SGM stigma and mental health to inform our work. When constructing our team, we sought inclusion of individuals with diverse sexual orientations and gender identities, alongside interdisciplinary expertise in mental health, SGM health disparities, online survey research, epidemiology, qualitative research, and latent variable modeling.

In addition, all study activities are guided by a Community Advisory Board (CAB) consisting of up to 10 diverse members from the study’s target population. CAB members are recruited on an ongoing basis through email listservs from rural SGM-serving organizations, geo-targeted advertisements on social networking websites, participants of the current study, and – when necessary – participants of relevant studies external to the current study. Potentially interested candidates complete a brief application, with a subsequent internal review and interview process used for selection. Geographical location, gender identity, sexual orientation, and race/ethnicity are factors included in evaluation to ensure diversity and equitable representation of rural populations within the US. CAB members serve a 12-month term with possible contract renewal. CAB members are responsible for evaluating study design, recruitment strategies, recruitment materials, and survey items. The CAB meets quarterly, for a total of four meetings per study year. Compensation for CAB members includes $100 per hour for services and meetings.

### Participants

Study participants will include up to 2,500 adults aged 18 + residing in rural counties and small metropolitan areas (< 250,000 population) across the following groups: up to 1,000 cisgender sexual minority persons (up to *N* = 500 each cisgender women and cisgender men); and up to 1,500 gender minority persons (up to *N* = 500 persons who were assigned male at birth and identify as a woman, female, and/or transfeminine; up to *N* = 500 persons who were assigned female at birth and identify as a man, male, and/or transmasculine; and up to *N* = 500 persons who identify as some other gender, including non-binary, gender non-conforming, and/or agender regardless of sex assigned at birth). We define cisgender as those whose current gender identity aligns with conventional assumptions of their sex assigned at birth. Inclusion criteria across these groups is shown in Table 1. To explore differences by race/ethnicity, we aim to enroll racial/ethnic minority persons at a level that is, at minimum, representative of the national demographics of the rural US according to 2020 census estimates, and will target recruitment strategies to platforms that have greater racial and ethnic minority representation and limit inclusion criteria as necessary based on ongoing enrollment progress.

**Table 1:** REALM cohort study inclusion criteria by participant type

Participant type	Inclusion criteria
Sexual minority persons	• At least 18 years old • Able to read English and/or Spanish • Willing to provide ≥ 2 means of contact and an emergency contact • Currently living in a rural county or small metropolitan area • Identifies gender as cisgender man or woman • Identifies sexuality as other than only heterosexual (specifically homosexual, including gay or lesbian; bisexual; pansexual; or queer)
Gender minority persons	• At least 18 years old • Able to read English and/or Spanish • Willing to provide ≥ 2 means of contact and an emergency contact • Currently living in a rural county or small metropolitan area • Identifies gender as something other than cisgender man or woman (specifically woman, female, transwoman, trans female or transfeminine when assigned male at birth; man, male, trans man, trans male, or transmasculine when assigned female at birth; or non-binary, gender nonconforming, or genderqueer)

### Sample size

We based our sample size calculations on limited previous studies capturing the prevalence of depression, suicidal ideation, and suicide attempts [1, 4, 7, 33, 63–71] – as well as distal minority stressors [34, 72, 73] – among SGM persons. For Aim 1, using conservative estimates, we expect a sample size of *N* = 500 in each SGM subgroup to provide a minimum of 98% power (normal approximation with continuity correction) to detect a 15% absolute difference in depression prevalence (estimated prevalence ratio of 1.6) between exposed and unexposed persons. We also expect this sample size to provide at minimum 98% power for a 15% absolute difference in both suicidal ideation and attempts. For the LCA, estimates of minimum required sample sizes in growth mixture models with 10 exposure measures ranged from 300 to 1,100 (in a six-class model with low class separation and 20% missing data) based on Monte Carlo simulations [74]. With an estimated 13 indicators of distal minority stressors, our total sample of 2,500 should therefore be adequate for estimating growth mixture models.

All participants will be followed for 12 months with a projected overall retention rate of 80% resulting in *N* = 400 participants in each SGM subgroup available for endline analyses. We will use the same retention procedures successfully implemented in a recent online prospective pilot study with gay men and other men who have sex with men that demonstrated retention rates of 98% at four months [75]. Though retention is not expected to vary by SGM subgroup, our Aim 2 power calculations account for expected variations in terms of the prevalence and incidence of depression and suicide attempts. Drawing on one general population study of depression incidence over a 24-month period [76], we have used a conservative estimate of average annual incidence of one-eighth of the estimated prevalence. We have also extrapolated from existing cross-sectional studies [7, 34, 66, 72, 73, 77, 78] to estimate that between 25–30% of participants will have additional exposures to distal minority stressors over the follow-up period.

Due to a lack of available studies, Aim 2 power calculations are based off 24-month rather than 12-month follow-up. We expect the projected sample size in each SGM subgroup followed through 24 months to provide a minimum of 78% power (normal approximation with continuity correction) to detect a 7% absolute difference in depression incidence (equivalent to an estimated incidence rate ratio of 4.5) between exposed and unexposed persons. Notably, these calculations are based on the sexual minority cisgender men subgroup of participants, who are predicted to have the lowest incidence of both depression and distal minority stressors. All other SGM subgroups are expected to have substantially higher power for depression incidence analyses: 84% for transgender men and transmasculine persons; 90% for sexual minority cisgender women; and 99% for transgender women and transfeminine persons.

### Recruitment

Study participants will primarily be recruited through geo-targeted advertisements on social networking websites and mobile applications, including Facebook, Instagram, Snapchat, YouTube, Tumblr, Reddit, X (formerly Twitter), TikTok, Tinder, and Grindr. In addition, a list of rural SGM-serving organizations will be compiled and asked to promote the study both by sharing advertisements on their websites, social media pages, and email lists as well as by distributing recruitment materials during in-person SGM-focused events. Recruitment will also be conducted via referral from other participants, the CAB, and the study team. Individuals who click on ads will be referred to a brief introduction script and will complete a self-administered eligibility screening survey through a secure online platform. If they are found to be eligible based on the screener, potential participants will be emailed a unique link to complete informed consent and the baseline survey. Study materials – including the recruitment materials, screening survey, and questionnaires – will be available in both English and Spanish to allow for the recruitment of rural Spanish-speaking SGM populations.

Participants will be enrolled in the study only upon validation (e.g., ensuring that participants are non-fraudulent and are not survey bots). The validation process will occur after the completion of the baseline survey and will be comprised of both manual and automated procedures. These will include phone number verification, IP address location verification, zip code confirmation, time-stamp review, duplicate enrollment check, review of survey responses for mismatched answers on repeat questions, completion of the baseline survey within 28 days of receipt, and completion of at least 75% of the baseline survey and mental health questions. Participants who do not pass this validation process will be marked as ineligible and stopped from participating in further study activities.

### Data collection

Study surveys will be administered online through Alchemer, a HIPAA-compliant survey software platform that stores data in a cloud-based repository which can only be accessed by staff using individual logins and passwords. In addition to a baseline survey, all participants will be asked to complete repeated online surveys administered every three months for a total of 12 months of follow-up post-enrollment (at months 3, 6, 9, and 12). A three-month gap in assessments was chosen to give sufficient time for change to occur in exposure variables without compromising participant recall or retention, or overburdening the participants. Participants will have a total of 60 days to complete each survey from the first date of availability. Participants who miss a survey will be allowed to complete future surveys and will remain in the study unless they request to discontinue participation. Non-fraudulent participants will be compensated $20 in the form of an e-gift card for each completed survey.

Given the risk of mental illness in this population, and survey questions focused on mental health and suicide, procedures have been established to promote the safety of study participants. All participants will be provided with a mental health resource list at the completion of the survey. In addition, survey responses to the Columbia Suicide Severity Rating Scale (C-SSRS) [79] that endorse suicidal ideation with suicide intent or a suicide plan in the last three months will prompt an email alert from the survey software to study clinicians, who are trained in crisis management. Initial outreach will occur within one business day of the email alert to ensure that the participant is safe. Outreach will continue until six contact attempts have been made or the participant is reached, whichever occurs first. Study clinicians will also reach out, up to three times, to emergency contacts if other methods are unsuccessful. Once contacted, study clinicians will assess the participant’s level of risk and conduct safety planning, lethal means counseling, and/or connect the participant to a crisis hotline, as deemed appropriate by the clinical staff member.

### Measures

Quantitative data collection for Aims 1 and 2 will focus on sociodemographic information, mental health, substance use, suicidal behaviors, minority stressors, psychological processes, and other related risk and protective factors. All measures are described in Table 2, and further details regarding the primary outcomes and exposures are included below.

**Table 2:** REALM cohort study measures

Domain	Measure	Description
***Mental health***		
Depression	Patient Health Questionnaire-9 (PHQ-9) [80]	9 items on frequency of depressive symptoms (past 2 weeks)
Suicidal ideation and attempts	Electronic Columbia Suicide Severity Rating Scale (eC-SSRS) [79]	11 items on suicidal ideation and behaviors (lifetime^c^ and past 3 months)
Anxiety	General Anxiety Disorder-7 (GAD-7) [112]	7 items on frequency of anxiety symptoms (past 2 weeks)
Post-traumatic stress	Post-Traumatic Stress Disorder Checklist for DSM-5 (PCL-5) [113]	20 items on post-traumatic stress symptoms (past month)
Substance use	Alcohol, Smoking and Substance Involvement Screening Test (ASSIST-Lite) [114]	10 items on substance use and associated impairment (past 3 months)
Alcohol use	Alcohol Use Disorder Identification Test – Concise (AUDIT-C) [115]	3 items on hazardous alcohol use (past 3 months)
Cross-cutting symptoms	DSM-5 Level 1 Cross-Cutting Symptom Measure (DSM-XC) [109]	23 items that assess the presence and severity of 13 symptom domains (past 2 weeks)
Functioning	World Health Organization Disability Assessment Schedule (WHODAS 2.0) [116]	12 items on physical, emotional, and psychological functioning (past month)
Access to and use of services	Adapted from National Survey on Drug Use and Health (NSDUH) [117]	5 items on seeking mental health care (past 3 months)
***Minority stressors***		
Stigma and discrimination	LGBTQ-Related Stigma and Discrimination Scale [59]	15 items on perceived, anticipated, and enacted stigma due to sexual orientation and/or gender identity (lifetime^c^ and past 3 months)
Intersectional discrimination	Intersectional Day-to-Day Discrimination Index [89]	9 items on general experiences of discrimination (lifetime^c^ and past 3 months)
Childhood adversity	Adverse Childhood Experiences Questionnaire^c^ [90]	10 items on adverse experiences in the first 18 years of life
General stressors	Life Events List (LEL) [91]	22 items on stressful experiences in the past year^c^ (or past 3 months)
Internalized stigma	Revised Internalized Homophobia Scale (IHP-R)^a^ [118]	5 items on negative feelings about sexual orientation (past 3 months)
	Transgender Identity Survey^b^ [119]	18 items on negative feelings about gender identity (past 3 months)
***Psychological processes***		
Hopelessness	Brief Beck Hopelessness Scale (Brief H-Neg) [120]	2 items on hopes for the future
Perceived burdensomeness	Perceived Burdensomeness Scale [121]	6 items on perceived burdensomeness to others
***Other risk and protective factors***		
Pride	Transgender Identity Survey subscale;^b^ adapted for sexual minorities^a^ [119]	8 items on pride about sexual orientation and/or gender identity (past 3 months)
Social support	Multidimensional Scale of Perceived Social Support (MSPSS) [122]	12 items on perceived social support from family, friends, and significant others
Resilience	Brief Resilience Scale [123]	6 items assessing ability to recover from stress
Flourishing	Secure Flourish [124]	12 items on happiness/life satisfaction, health, meaning and purpose, character/virtue, close relationships, and financial/material stability
General religiosity	Adapted from Rosmarin et al. [125]	4 items on religious beliefs and identity
Organizational religiosity	Adapted from Brief Multidimensional Measure of Religiosity/Spirituality [126]	3 items on organized religious activities
Religious conflict	Adapted from Eden et al. [127]	4 items religious conflict due to sexual orientation and/or gender identity
Structural religious support	Adapted from Eden et al. [127]	2 items on structural support from religious community

^a^ Sexual minorities only

^b^ Gender minorities only

^c^ Measured at baseline only

Alongside included measures, study will also capture sociodemographic information including sexual orientation, gender identity, race/ethnicity, language, education, employment status, food insecurity, income, birth country, housing, incarceration history, gender congruence/presentation/medical affirmation, attraction, and sexual orientation/gender identity disclosure/concealment

#### Primary outcomes

The primary outcomes for this study are depression, suicidal ideation, and suicide attempts. Depression will be assessed using the Patient Health Questionnaire-9 (PHQ-9) [80] and suicidal ideation and attempts will be assessed using the electronic self-reported version of the C-SSRS [79]. The PHQ-9 and the CSSR-S are validated screening tools with clinical and research applications, and have previously been used with SGM populations [81–86]. Both are part of the Suicide Prevention Resource Center's Suicide Prevention Toolkit for Primary Care Practices [87]. These assessments are relatively brief, which is expected to improve response and completion rates in the study, and they can also be scaled such that they assess experiences since last assessment. Both assessments include scale scoring that is useful for determining potential severity of symptoms as a means of differentiating risks or monitoring changes over time.

#### Primary exposures

The primary exposures for this study are distal minority stressors including stigma, discrimination, and traumatic experiences. Stigma and discrimination will be assessed using the enacted stigma subscale from the LGBTQ-Related Stigma and Discrimination Scale, developed and psychometrically evaluated by our team among SGM populations domestically and internationally [34, 59, 88]. Participants report whether they have experienced (ever and within the past three months) discrimination, violence, and harassment because of their sexual orientation and/or gender identity. We will also include the Intersectional Day-to-Day Discrimination Index, which assesses general experiences (ever and within the past three months) of discrimination, microaggressions, and harassment and has been validated for use in diverse SGM populations [89]. Traumatic experiences will be assessed using the Adverse Childhood Experiences Questionnaire [90], which evaluates childhood trauma experienced prior to age 18, and the Life Events List [91], which evaluates general stressors experienced within the past year (or the past three months at follow-up). Across these measures, specific indicators will be explored and selected for use in subsequent data analysis.

### Data analysis

For Aim 1, we will use latent class analysis (LCA) to explore how clusters of stigma, discrimination, and traumatic experiences (i.e., distal minority stressors) may be differentially associated with mental health-related outcomes among SGM persons at baseline. LCA is a person-centered analytic approach that allows for the identification of underlying classes of participants based on their responses to observed indicator variables [92]. Using the indicators described above to capture distal minority stressors, we will perform class enumeration by testing models with an increasing number of classes. The best fitting model will be selected using fit indices such as the Akaike Information Criteria, Bayesian Information Criteria, Vuong-Lo-Mendell-Rubin Likelihood Ratio Test, and entropy, and further evaluated based on theoretical interpretability [93]. All initial analyses will be conducted separately by the five SGM subgroups (sexual minority cisgender women, sexual minority cisgender men, transgender women and transfeminine persons, transgender men and transmasculine persons, and non-binary persons). Various modeling approaches will be examined to address potential effect modification by sexual orientation or gender identity within these five subgroups, and multigroup alignment optimization will be used to assess measurement invariance. If the latent classes are similar across subgroups, we will combine and repeat the effect modification and optimization analysis for gender minority versus cisgender participants. Finally, we will use the Bolck, Croon, and Hagenaars latent class regression approach to assess associations between class membership and prevalent depression, suicidal ideation, and suicide attempts [94]. In addition, age and racial/ethnic identities will be included as covariates, with possible interactions with latent class.

For Aim 2, we will use Kaplan–Meier and Cox proportional regression models to describe fixed and time-varying factors/classes and their independent associations with the incidence of self-reported symptoms of moderate or severe depression, suicidal ideation, or suicide attempts during follow-up. Model findings will be reported as adjusted hazards ratios with 95% CIs. Similar to Aim 1 analyses, we will first conduct stratified incidence analyses by SGM subgroups, with subsequent analyses exploring stratification by age and racial/ethnic identities. Guided by our theoretical framework, which combines IPTS with minority stress models, we will also implement mediation models within classes using Structural Equation Mixture Modeling [95, 96]. Secondary analyses will explore recurrent or persistent (as opposed to incident) depression and suicidal ideation using a combination of approaches that can address time-varying, repeated outcomes or changing severity of outcomes [97]. We will also conduct analyses with and without controlling for prior/lifetime reports of major depression, suicidal ideation, and/or suicide attempts, and examine the effects of potential temporality/lag for time-varying exposures that may have overlapped during periods of reported symptoms.

## Discussion

REALM, as a prospective cohort study focused on both sexual minority and gender minority adults living in rural areas of the US, presents a critical opportunity to better understand how minority stress contributes to adverse mental health outcomes and suicidal behaviors among populations that remain underrepresented in research and programs in 2024. By recruiting a large and heterogeneous sample of rural SGM adults that can be followed prospectively, REALM aims not only to identify patterns of exposure to distal minority stressors (e.g., stigma, discrimination, and traumatic experiences) that influence both the prevalence and incidence of depression, suicidal ideation, and suicide attempts through multiple complex pathways, but also to integrate factors from suicide-focused theories, consider key protective factors, and explore diversity within SGM communities. Given the lack of established mechanisms for reaching rural SGM populations, this study will leverage online methods to recruit and retain up to 2,500 persons.

REALM is poised to fill a sizeable gap in evidence around mental health among SGM adults living in rural areas, with implications for the development of responsive interventions. It is important to highlight that for gender minority populations, specifically, prevalence data on mental health conditions and suicidal behaviors are lacking even in urban settings. While newer initiatives like the 2015 and 2022 US Transgender Surveys [25] and the Leading Innovation for Transgender Women’s Health and Empowerment (LITE) Cohort Study [98] are beginning to fill these gaps, they are either cross-sectional, urban, or predominantly focused on sexual health. As such, REALM will provide complementary rather than overlapping information to these important initiatives.

There are several potential drivers of this limited focus on mental health among rural SGM adults. Most importantly, these populations may be harder to identify and reach given rural–urban differences related to identity disclosure, socialization and congregation, and the availability of SGM-specific health services and other resources [40, 99–101]. Our study seeks to address this challenge by drawing upon established methods of online recruitment for SGM populations that can be geotargeted, with the potential to combine these efforts with advertising and recruitment in physical spaces guided by input from our CAB. Secondly, collecting information on suicidal ideation and behaviors has ethical implications given the need to ensure participants’ safety, and represents a greater investment from both researchers and participants in the study. For SGM populations, this can be particularly fraught as there are concerns about unintentionally outing someone when talking to an emergency contact, whether services will be safe or affirming, and the potential for structural stigma to be enacted through different institutions that would be called upon to help ensure safety (e.g., police force, healthcare providers). The REALM CAB, representation of lived experience among the research team, interdisciplinary collaboration, and careful and intensive planning were critical to ensuring that rigorous safety procedures, that take these concerns into consideration, could be established. These resources can help guide future studies that face similar challenges.

There are several possible limitations to REALM that must be acknowledged. First, there is potential for this study to fall short of the targeted enrollment sizes by stratified subgroup. We will adjust recruitment strategies based on real-time monitoring of recruitment outcomes to ensure representation across cohorts of SGM adults with different identities and by race/ethnicity. Second, online procedures may pose challenges to retention [102], although we have shown high retention in prior virtual cohort studies that have employed similar retention procedures and therefore do not anticipate this issue [103–105]. Third, previous research has shown that online study procedures can result in biases in both enrollment and retention compared to the general population [102, 106]. However, the online study processes we have developed over the past several years have resulted in study samples that are approximately consistent with general US population estimates in terms of race/ethnicity and urbanicity [107–109]. Lastly, engagement in study procedures may systematically vary by the primary exposures and outcomes of interest. We will examine the potential for these biases as the study is being conducted and employ statistical approaches to address systematic biases in missing data if necessary [110, 111].

Understanding the processes through which minority stressors shape the mental health of diverse SGM persons living in rural areas of the US is critical to building outreach approaches and preventive interventions that can engage understudied groups of SGM adults who experience health disparities and limited access to affirming and effective resources. As policy landscapes change throughout the US, and structural stigma and violence continue to shape the lived realities of SGM communities, efforts to protect and promote mental health are both timely and essential. By being grounded in minority stress and suicide theories, using established online and diversified recruitment methods to reach underprioritized populations, ensuring enrollment of SGM adults with diverse identities, cultivating and nurturing a diverse study team, and collaborating closely with a CAB with relevant lived experience, REALM will work to address key gaps in knowledge on the processes that produce poor mental health among rural SGM adults in the US. In doing so, REALM will generate strategies for how best to support well-being among these vulnerable populations. Most immediately, this will include leveraging REALM’s online sample to inform the development of a novel technology-delivered depression support and suicide prevention intervention, with participatory processes used to help ensure that content and delivery strategies are tailored, acceptable, and relevant to the target population. The cohort data set, by being made accessible in a National Institute of Mental Health online repository, will also be available to support other secondary analyses that can further inform interventions to promote mental health and well-being for SGM adults in rural areas.

## Data Availability

The data associated with this study will be uploaded to the National Institute of Mental Health Data Archive for public use [128].

## Abbreviations

SGM: Sexual and gender minority
LGBTQ: Lesbian, gay, bisexual, transgender, and queer
US: United States
IPTS: Interpersonal-Psychological Theory of Suicidal Behavior
REALM: Rural Exploration and Approaches for LGBTQ + Mental Health
CAB: Community Advisory Board
PHQ-9: Patient Health Questionnaire-9
C-SSRS: Columbia Suicide Severity Rating Scale
LCA: Latent class analysis

## References

[1] King M, Semlyen J, Tai SS, Killaspy H, Osborn D, Popelyuk D, et al. A systematic review of mental disorder, suicide, and deliberate self harm in lesbian, gay and bisexual people. BMC Psychiatry. 2008;8:70. 18706118 10.1186/1471-244X-8-70 PMC2533652

[2] Marchi M, Arcolin E, Fiore G, Travascio A, Uberti D, Amaddeo F, et al. Self-harm and suicidality among LGBTIQ people: a systematic review and meta-analysis. Int Rev Psychiatry. 2022;34:240–56. 36151841 10.1080/09540261.2022.2053070

[3] Pinna F, Paribello P, Somaini G, Corona A, Ventriglio A, Corrias C, et al. Mental health in transgender individuals: a systematic review. Int Rev Psychiatry Abingdon Engl. 2022;34:292–359. 10.1080/09540261.2022.2093629 36151828

[4] Medley G LR Bose J, Cribb DS, Kroutil LA, McHenry G. Sexual Orientation and Estimates of Adult Substance Use and Mental Health: Results from the 2015 National Survey on Drug Use and Health. NSDUH Data Review. 2016;1-54.

[5] Substance Abuse and Mental Health Services Administration. Lesbian, gay, and bisexual behavioral health: Results from the 2021 and 2022 National Surveys on Drug Use and Health (SAMHSA Publication No. PEP23-07-01-001). Center for Behavioral Health Statistics and Quality, Substance Abuse and Mental Health Services Administration. https://www.samhsa.gov/data/report/LGB-Behavioral-Health-Report-2021-2022.

[6] Capistrant BD, Nakash O. Suicide Risk for Sexual Minorities in Middle and Older Age: Evidence From the National Survey on Drug Use and Health. Am J Geriatr Psychiatry. 2019;27:559–63. 30770188 10.1016/j.jagp.2018.12.023

[7] Downing JM, Przedworski JM. Health of Transgender Adults in the U.S., 2014–2016. Am J Prev Med. 2018;55:336–44. 10.1016/j.amepre.2018.04.045 30031640

[8] Rosner B, Neicun J, Yang JC, Roman-Urrestarazu A. Substance use among sexual minorities in the US – Linked to inequalities and unmet need for mental health treatment? Results from the National Survey on Drug Use and Health (NSDUH). J Psychiatr Res. 2021;135:107–18. 33472121 10.1016/j.jpsychires.2020.12.023

[9] Kidd JD, Tettamanti NA, Kaczmarkiewicz R, Corbeil TE, Dworkin JD, Jackman KB, et al. Prevalence of substance use and mental health problems among transgender and cisgender U.S. adults: Results from a national probability sample. Psychiatry Res. 2023;326:115339. 10.1016/j.psychres.2023.115339 PMC10528335 37429172

[10] Clark KA, Mays VM, Arah OA, Kheifets LI, Cochran SD. Sexual Orientation Differences in Lethal Methods Used in Suicide: Findings From the National Violent Death Reporting System. Arch Suicide Res. 2022;26:548–64. 32897837 10.1080/13811118.2020.1811181 PMC7937759

[11] Plöderl M, Tremblay P. Mental health of sexual minorities. A systematic review Int Rev Psychiatry. 2015;27:367–85. 26552495 10.3109/09540261.2015.1083949

[12] Hirsch JK, Cohn TJ, Rowe CA, Rimmer SE. Minority Sexual Orientation, Gender Identity Status and Suicidal Behavior: Serial Indirect Effects of Hope, Hopelessness and Depressive Symptoms. Int J Ment Health Addict. 2017;15:260–70.

[13] Meyer IH. Prejudice, Social Stress, and Mental Health in Lesbian, Gay, and Bisexual Populations: Conceptual Issues and Research Evidence. Psychol Bull. 2003;129:674–97. 12956539 10.1037/0033-2909.129.5.674 PMC2072932

[14] Hendricks ML, Testa RJ. A conceptual framework for clinical work with transgender and gender nonconforming clients: An adaptation of the Minority Stress Model. Prof Psychol Res Pract. 2012;43:460–7.

[15] Brooks VR. Minority stress and lesbian women. Lexington: Lexington Books; 1981.

[16] Link BG, Phelan JC. Conceptualizing Stigma. Annu Rev Sociol. 2001;27:363–85.

[17] Tebbe EA, Moradi B. Suicide risk in trans populations: An application of minority stress theory. J Couns Psychol. 2016;63:520–33. 27089059 10.1037/cou0000152

[18] Horwitz AG, Berona J, Busby DR, Eisenberg D, Zheng K, Pistorello J, et al. Variation in Suicide Risk among Subgroups of Sexual and Gender Minority College Students. Suicide Life Threat Behav. 2020;50:1041–53. 32291833 10.1111/sltb.12637 PMC7981781

[19] Staples JM, Neilson EC, Bryan AEB, George WH. The Role of Distal Minority Stress and Internalized Transnegativity in Suicidal Ideation and Nonsuicidal Self-Injury Among Transgender Adults. J Sex Res. 2018;55:591–603. 29148860 10.1080/00224499.2017.1393651

[20] McNeil J, Ellis SJ, Eccles FJR. Suicide in trans populations: A systematic review of prevalence and correlates. Psychol Sex Orientat Gend Divers. 2017;4:341–53.

[21] Plöderl M, Sellmeier M, Fartacek C, Pichler E-M, Fartacek R, Kralovec K. Explaining the Suicide Risk of Sexual Minority Individuals by Contrasting the Minority Stress Model with Suicide Models. Arch Sex Behav. 2014;43:1559–70. 24573399 10.1007/s10508-014-0268-4

[22] Fulginiti A, Goldbach JT, Mamey MR, Rusow J, Srivastava A, Rhoades H, et al. Integrating Minority Stress Theory and the Interpersonal Theory of Suicide among Sexual Minority Youth Who Engage Crisis Services. Suicide Life Threat Behav. 2020;50:601–16. 32048340 10.1111/sltb.12623

[23] Klonsky ED, Saffer BY, Bryan CJ. Ideation-to-action theories of suicide: a conceptual and empirical update. Curr Opin Psychol. 2018;22:38–43. 30122276 10.1016/j.copsyc.2017.07.020

[24] Joiner T. Why People Die by Suicide. Boston, MA: Harvard University Press; 2005.

[25] James SE, Herman JL, Rankin S, Keisling M, Mottet L, Anafi M. The Report of the 2015 U.S. Transgender Survey. Washington, DC: National Center for Transgender Equality; 2016.

[26] Friedman MS, Marshal MP, Guadamuz TE, Wei C, Wong CF, Saewyc E, et al. A Meta-Analysis of Disparities in Childhood Sexual Abuse, Parental Physical Abuse, and Peer Victimization Among Sexual Minority and Sexual Nonminority Individuals. Am J Public Health. 2011;101:1481–94. 21680921 10.2105/AJPH.2009.190009 PMC3134495

[27] Rothman EF, Exner D, Baughman AL. The prevalence of sexual assault against people who identify as gay, lesbian, or bisexual in the United States: a systematic review. Trauma Violence Abuse. 2011;12:55–66. 21247983 10.1177/1524838010390707 PMC3118668

[28] Langenderfer-Magruder L, Walls NE, Kattari SK, Whitfield DL, Ramos D. Sexual Victimization and Subsequent Police Reporting by Gender Identity Among Lesbian, Gay, Bisexual, Transgender, and Queer Adults. Violence Vict. 2016;31:320–31. 26831853 10.1891/0886-6708.VV-D-14-00082

[29] Testa RJ. Suicidal ideation in transgender people: Gender minority stress and interpersonal theory factors. J Abnorm Psychol. 2017;126:125–36. 27831708 10.1037/abn0000234

[30] Casant J, Helbich M. Inequalities of Suicide Mortality across Urban and Rural Areas: A Literature Review. Int J Environ Res Public Health. 2022;19:2669. 35270369 10.3390/ijerph19052669 PMC8909802

[31] Jenkins WD, Walters S, Phillips G, Green K, Fenner E, Bolinski R, et al. Stigma, Mental Health, and Health care Use Among Rural Sexual and Gender Minority Individuals. Health Educ Behav Off Publ Soc Public Health Educ. 2024;51:477–89. 10.1177/10901981221120393 PMC10064479 36036544

[32] Dyar C, Morgan E. Rural and urban differences in disparities in substance use and substance use disorders affecting sexual minority populations. J Rural Health. 2024;40:542–56. 38112341 10.1111/jrh.12816 PMC11187699

[33] Horvath KJ, Iantaffi A, Swinburne-Romine R, Bockting W. A comparison of mental health, substance use, and sexual risk behaviors between rural and non-rural transgender persons. J Homosex. 2014;61:1117–30. 24380580 10.1080/00918369.2014.872502 PMC4301267

[34] Stahlman S, Sanchez TH, Sullivan PS, Ketende S, Lyons C, Charurat ME, et al. The Prevalence of Sexual Behavior Stigma Affecting Gay Men and Other Men Who Have Sex with Men Across Sub-Saharan Africa and in the United States. JMIR Public Health Surveill. 2016;2: e35. 27460627 10.2196/publichealth.5824 PMC4978863

[35] Kauth MR, Barrera TL, Denton FN, Latini DM. Health Differences Among Lesbian, Gay, and Transgender Veterans by Rural/Small Town and Suburban/Urban Setting. LGBT Health. 2017;4:194–201. 28430020 10.1089/lgbt.2016.0213

[36] Goldbach JT, Parra LA, O’Brien RP, Rhoades H, Schrager SM. Explaining behavioral health differences in urban and rural sexual minority adolescents: A longitudinal investigation of minority stress in a diverse national sample of sexual minority adolescents: A longitudinal investigation of minority stress in a diverse national sample of sexual minority adolescents. J Rural Health. 2023;39:262–71. 35977886 10.1111/jrh.12706 PMC9771913

[37] Kaplan SC, Butler RM, Devlin EA, Testa RJ, Horenstein A, Swee MB, et al. Rural living environment predicts social anxiety in transgender and gender nonconforming individuals across Canada and the United States. J Anxiety Disord. 2019;66: 102116. 31357038 10.1016/j.janxdis.2019.102116

[38] Swank E, Frost DM, Fahs B. Rural location and exposure to minority stress among sexual minorities in the United States. Psychol Sex. 2012;3:226–43.

[39] Irwin JA, Coleman JD, Fisher CM, Marasco VM. Correlates of suicide ideation among LGBT Nebraskans. J Homosex. 2014;61:1172–91. 24344775 10.1080/00918369.2014.872521

[40] Whitehead J, Shaver J, Stephenson R. Outness, Stigma, and Primary Health Care Utilization among Rural LGBT Populations. PLoS ONE. 2016;11: e0146139. 26731405 10.1371/journal.pone.0146139 PMC4701471

[41] Blosnich JR, De Luca S, Lytle MC, Brownson C. Questions of faith: Religious affiliations and suicidal ideation among sexual minority young adults. Suicide Life Threat Behav. 2020;50:1158–66. 32744388 10.1111/sltb.12679 PMC10334798

[42] Clarke K, Saville N, Shrestha B, Costello A, King M, Manandhar D, et al. Predictors of psychological distress among postnatal mothers in rural Nepal: A cross-sectional community-based study. J Affect Disord. 2014;156:76–86. 24370265 10.1016/j.jad.2013.11.018 PMC3969296

[43] Chalfant HP, Heller PL. Rural/Urban versus Regional Differences in Religiosity. Rev Relig Res. 1991;33:76–86.

[44] Rigg KK, Monnat SM. Urban vs. rural differences in prescription opioid misuse among adults in the United States: Informing region specific drug policies and interventions. Int J Drug Policy. 2015;26:484–91. 10.1016/j.drugpo.2014.10.001 PMC4397122 25458403

[45] Wu A, Wang J-Y, Jia C-X. Religion and Completed Suicide: a Meta-Analysis. PLoS ONE. 2015;10: e0131715. 26110867 10.1371/journal.pone.0131715 PMC4482518

[46] Lawrence RE, Oquendo MA, Stanley B. Religion and Suicide Risk: a systematic review. Arch Suicide Res Off J Int Acad Suicide Res. 2016;20:1–21. 10.1080/13811118.2015.1004494 PMC7310534 26192968

[47] Lytle MC, Blosnich JR, De Luca SM, Brownson C. Association of Religiosity With Sexual Minority Suicide Ideation and Attempt. Am J Prev Med. 2018;54:644–51. 29550162 10.1016/j.amepre.2018.01.019 PMC10782832

[48] Lyons BH, Walters ML, Jack SPD, Petrosky E, Blair JM, Ivey-Stephenson AZ. Suicides Among Lesbian and Gay Male Individuals: Findings From the National Violent Death Reporting System. Am J Prev Med. 2019;56:512–21. 30898221 10.1016/j.amepre.2018.11.012 PMC6886000

[49] Jordan JT, McNiel DE. Characteristics of persons who die on their first suicide attempt: results from the National Violent Death Reporting System. Psychol Med. 2020;50:1390–7. 31217042 10.1017/S0033291719001375

[50] Miranda-Mendizábal A, Castellví P, Parés-Badell O, Almenara J, Alonso I, Blasco MJ, et al. Sexual orientation and suicidal behaviour in adolescents and young adults: systematic review and meta-analysis. Br J Psychiatry J Ment Sci. 2017;211:77–87. 10.1192/bjp.bp.116.196345 28254960

[51] Meyer IH, Russell ST, Hammack PL, Frost DM, Wilson BDM. Minority stress, distress, and suicide attempts in three cohorts of sexual minority adults: A U.S. probability sample. PLOS ONE. 2021;16:e0246827. 10.1371/journal.pone.0246827 PMC7928455 33657122

[52] Bowleg L. The Problem With the Phrase Women and Minorities: Intersectionality—an Important Theoretical Framework for Public Health. Am J Public Health. 2012;102:1267–73. 22594719 10.2105/AJPH.2012.300750 PMC3477987

[53] Bowleg L, Malekzadeh AN, AuBuchon KE, Ghabrial MA, Bauer GR. Rare exemplars and missed opportunities: Intersectionality within current sexual and gender diversity research and scholarship in psychology. Curr Opin Psychol. 2023;49: 101511. 36586378 10.1016/j.copsyc.2022.101511 PMC10787324

[54] English D, Rendina HJ, Parsons JT. The Effects of Intersecting Stigma: A Longitudinal Examination of Minority Stress, Mental Health, and Substance Use among Black, Latino, and Multiracial Gay and Bisexual Men. Psychol Violence. 2018;8:669–79. 30881729 10.1037/vio0000218 PMC6415673

[55] Xavier Hall CD, Harris R, Burns P, Girod C, Yount KM, Wong FY. Utilizing Latent Class Analysis to Assess the Association of Intersectional Stigma on Mental Health Outcomes Among Young Adult Black, Indigenous, and Sexual Minority Women of Color. LGBT Health. 2023;10:463–70. 36951670 10.1089/lgbt.2022.0083 PMC10468552

[56] Walubita T, Beccia AL, Boama-Nyarko E, Ding EY, Ferrucci KA, Jesdale BM. Complicating Narratives of Sexual Minority Mental Health: An Intersectional Analysis of Frequent Mental Distress at the Intersection of Sexual Orientation, Gender Identity, and Race/Ethnicity. LGBT Health. 2022;9:161–8. 35180360 10.1089/lgbt.2021.0099

[57] Borgogna NC, McDermott RC, Aita SL, Kridel MM. Anxiety and depression across gender and sexual minorities: Implications for transgender, gender nonconforming, pansexual, demisexual, asexual, queer, and questioning individuals. Psychol Sex Orientat Gend Divers. 2019;6:54–63.

[58] Murray SM, Wiginton JM, Xue Q-L, Dibble K, Sanchez T, Kane JC, et al. Measuring Sexual Behavior Stigma Among Cisgender Men Who Have Sex With Men: An Assessment of Cross-Country Measurement Invariance. Stigma Health. 2023;9:349–61. 39185350 10.1037/sah0000443 PMC11343079

[59] Augustinavicius JL, Baral SD, Murray SM, Jackman K, Xue Q-L, Sanchez TH, et al. Characterizing Cross-Culturally Relevant Metrics of Stigma Among Men Who Have Sex With Men Across 8 Sub-Saharan African Countries and the United States. Am J Epidemiol. 2020;189:690–7. 31942619 10.1093/aje/kwz270 PMC7608078

[60] Wiginton JM, Maksut JL, Scheim AI, Zlotorzynska M, Sanchez TH, Baral SD. Intersecting Sexual Behavior and Gender Identity Stigmas Among Transgender Women in the United States: Burden and Associations with Sexual Health. AIDS Behav. 2023;27:3064–79. 36952112 10.1007/s10461-023-04028-w PMC10034890

[61] Wiginton JM, Murray SM, Baral SD, Sanchez TH. Targeted Violence as a Risk Factor for Posttraumatic Stress Disorder Among Cisgender Gay, Bisexual, and Other Men Who have Sex with Men in the United States. J Interpers Violence. 2023;38:9739–64. 37118946 10.1177/08862605231169755 PMC10527206

[62] Wiginton JM, Murray SM, Anderson BJ, Sey K, Ma Y, Flynn CP, et al. Sexual behavior stigma among cisgender gay, bisexual, and other men who have sex with men in nine NHBS cities across the United States: Burden and associations with PrEP continuum and HIV care continuum outcomes. Stigma Health. 2024; Advance online publication. 10.1037/sah0000495. 10.1037/sah0000495 PMC11810123 39935519

[63] Marshal MP, Dietz LJ, Friedman MS, Stall R, Smith HA, McGinley J, et al. Suicidality and depression disparities between sexual minority and heterosexual youth: a meta-analytic review. J Adolesc Health. 2011;49:115–23. 21783042 10.1016/j.jadohealth.2011.02.005 PMC3649127

[64] Cochran SD, Mays VM, Sullivan JG. Prevalence of mental disorders, psychological distress, and mental health services use among lesbian, gay, and bisexual adults in the United States. J Consult Clin Psychol. 2003;71:53–61. 12602425 10.1037//0022-006x.71.1.53 PMC4197971

[65] Ross LE, Salway T, Tarasoff LA, MacKay JM, Hawkins BW, Fehr CP. Prevalence of Depression and Anxiety Among Bisexual People Compared to Gay, Lesbian, and Heterosexual Individuals: A Systematic Review and Meta-Analysis. J Sex Res. 2018;55:435–56. 29099625 10.1080/00224499.2017.1387755

[66] Bockting WO, Miner MH, Swinburne Romine RE, Hamilton A, Coleman E. Stigma, mental health, and resilience in an online sample of the US transgender population. Am J Public Health. 2013;103:943–51. 23488522 10.2105/AJPH.2013.301241 PMC3698807

[67] Owen-Smith AA, Gerth J, Sineath RC, Barzilay J, Becerra-Culqui TA, Getahun D, et al. Association Between Gender Confirmation Treatments and Perceived Gender Congruence, Body Image Satisfaction, and Mental Health in a Cohort of Transgender Individuals. J Sex Med. 2018;15:591–600. 29463478 10.1016/j.jsxm.2018.01.017 PMC5882508

[68] Mays VM, Cochran SD. Mental health correlates of perceived discrimination among lesbian, gay, and bisexual adults in the United States. Am J Public Health. 2001;91:1869–76. 11684618 10.2105/ajph.91.11.1869 PMC1446893

[69] Bostwick WB, Boyd CJ, Hughes TL, West BT, McCabe SE. Discrimination and mental health among lesbian, gay, and bisexual adults in the United States. Am J Orthopsychiatry. 2014;84:35–45. 24826824 10.1037/h0098851 PMC4144327

[70] Kerridge BT, Pickering RP, Saha TD, Ruan WJ, Chou SP, Zhang H, et al. Prevalence, sociodemographic correlates and DSM-5 substance use disorders and other psychiatric disorders among sexual minorities in the United States. Drug Alcohol Depend. 2017;170:82–92. 27883948 10.1016/j.drugalcdep.2016.10.038 PMC6310166

[71] Nuttbrock L, Bockting W, Rosenblum A, Hwahng S, Mason M, Macri M, et al. Gender abuse and major depression among transgender women: a prospective study of vulnerability and resilience. Am J Public Health. 2014;104:2191–8. 24328655 10.2105/AJPH.2013.301545 PMC4202964

[72] Lee C, Oliffe JL, Kelly MT, Ferlatte O. Depression and Suicidality in Gay Men: Implications for Health Care Providers. Am J Mens Health. 2017;11:910–9. 28103765 10.1177/1557988316685492 PMC5675322

[73] Stahlman S, Hargreaves JR, Sprague L, Stangl AL, Baral SD. Measuring Sexual Behavior Stigma to Inform Effective HIV Prevention and Treatment Programs for Key Populations. JMIR Public Health Surveill. 2017;3: e23. 28446420 10.2196/publichealth.7334 PMC5425775

[74] Kim S-Y. Determining the Number of Latent Classes in Single- and Multi-Phase Growth Mixture Models. Struct Equ Model Multidiscip J. 2014;21:263–79. 10.1080/10705511.2014.882690 PMC3979564 24729675

[75] Sullivan PS, Driggers R, Stekler JD, Siegler A, Goldenberg T, McDougal SJ, et al. Usability and Acceptability of a Mobile Comprehensive HIV Prevention App for Men Who Have Sex With Men: A Pilot Study. JMIR MHealth UHealth. 2017;5: e7199. 10.2196/mhealth.7199 PMC5364322 28279949

[76] Meng X, Brunet A, Turecki G, Liu A, D’Arcy C, Caron J. Risk factor modifications and depression incidence: a 4-year longitudinal Canadian cohort of the Montreal Catchment Area Study. BMJ Open. 2017;7: e015156. 28601831 10.1136/bmjopen-2016-015156 PMC5734363

[77] Mustanski BS, Garofalo R, Emerson EM. Mental health disorders, psychological distress, and suicidality in a diverse sample of lesbian, gay, bisexual, and transgender youths. Am J Public Health. 2010;100:2426–32. 20966378 10.2105/AJPH.2009.178319 PMC2978194

[78] Sanchez T, Finlayson T, Murrill C, Guilin V, Dean L. Risk behaviors and psychosocial stressors in the new york city house ball community: a comparison of men and transgender women who have sex with men. AIDS Behav. 2010;14:351–8. 19763812 10.1007/s10461-009-9610-6

[79] Posner K, Brown GK, Stanley B, Brent DA, Yershova KV, Oquendo MA, et al. The Columbia-Suicide Severity Rating Scale: initial validity and internal consistency findings from three multisite studies with adolescents and adults. Am J Psychiatry. 2011;168:1266–77. 22193671 10.1176/appi.ajp.2011.10111704 PMC3893686

[80] Spitzer RL, Kroenke K, Williams JB. Validation and utility of a self-report version of PRIME-MD: the PHQ primary care study. Primary Care Evaluation of Mental Disorders. Patient Health Questionnaire. JAMA. 1999;282:1737–44. 10.1001/jama.282.18.1737 10568646

[81] Hickson F, Davey C, Reid D, Weatherburn P, Bourne A. Mental health inequalities among gay and bisexual men in England, Scotland and Wales: a large community-based cross-sectional survey. J Public Health Oxf Engl. 2017;39:266–73. 10.1093/pubmed/fdw021 27118380

[82] Lowe B, Unutzer J, Callahan CM, Perkins AJ, Kroenke K. Monitoring depression treatment outcomes with the patient health questionnaire-9. Med Care. 2004;42:1194–201. 15550799 10.1097/00005650-200412000-00006

[83] Lowe B, Kroenke K, Herzog W, Grafe K. Measuring depression outcome with a brief self-report instrument: sensitivity to change of the Patient Health Questionnaire (PHQ-9). J Affect Disord. 2004;81:61–6. 15183601 10.1016/S0165-0327(03)00198-8

[84] Rimes KA, Broadbent M, Holden R, Rahman Q, Hambrook D, Hatch SL, et al. Comparison of Treatment Outcomes Between Lesbian, Gay, Bisexual and Heterosexual Individuals Receiving a Primary Care Psychological Intervention. Behav Cogn Psychother. 2018;46:332–49. 28978366 10.1017/S1352465817000583

[85] Ross LE, Bauer GR, MacLeod MA, Robinson M, MacKay J, Dobinson C. Mental Health and Substance Use among Bisexual Youth and Non-Youth in Ontario. Canada PLOS ONE. 2014;9: e101604. 25111292 10.1371/journal.pone.0101604 PMC4128599

[86] Bazargan M, Galvan F. Perceived discrimination and depression among low-income Latina male-to-female transgender women. BMC Public Health. 2012;12:663. 22894701 10.1186/1471-2458-12-663 PMC3497862

[87] Western Interstate Commission for Higher Education Mental Health Program (WICHE MHP), Suicide Prevention Resource Center (SPRC). Suicide prevention toolkit for primary care practices: A guide for primary care providers and medical practice managers. Boulder: WICHE MHP & SPRC; 2017.

[88] Maksut JL, Sanchez TH, Wiginton JM, Scheim AI, Logie CH, Zlotorzynska M, et al. Gender identity and sexual behavior stigmas, severe psychological distress, and suicidality in an online sample of transgender women in the United States. Ann Epidemiol. 2020;52:15–22. 32768521 10.1016/j.annepidem.2020.07.020 PMC7725857

[89] Scheim AI, Bauer GR. The Intersectional Discrimination Index: Development and validation of measures of self-reported enacted and anticipated discrimination for intercategorical analysis. Soc Sci Med. 2019;226:225–35. 30674436 10.1016/j.socscimed.2018.12.016

[90] Felitti VJ, Anda RF, Nordenberg D, Williamson DF, Spitz AM, Edwards V, et al. Relationship of childhood abuse and household dysfunction to many of the leading causes of death in adults. The Adverse Childhood Experiences (ACE) Study. Am J Prev Med. 1998;14:245–58. 10.1016/s0749-3797(98)00017-8 9635069

[91] Cohen S, Tyrrell DA, Smith AP. Psychological stress and susceptibility to the common cold. N Engl J Med. 1991;325:606–12. 1713648 10.1056/NEJM199108293250903

[92] Collins LM, Lanza ST. Latent class and latent transition analysis: With applications in the social, behavioral, and health sciences. Hoboken: John Wiley & Sons; 2010.

[93] Nylund KL, Asparouhov T, Muthén BO. Deciding on the Number of Classes in Latent Class Analysis and Growth Mixture Modeling: A Monte Carlo Simulation Study. Struct Equ Model Multidiscip J. 2007;14:535–69.

[94] Asparouhov T, Muthén B. Auxiliary Variables in Mixture Modeling: Using the BCH Method in Mplus to Estimate a Distal Outcome Model and an Arbitrary Secondary Model. MPlus Web notes. 2021;21:1–80.

[95] Vermunt JK, Magidson J. Structural Equation Models: Mixture Models. In: Encyclopedia of Statistics in Behavioral Science. Wiley; 2005. p. 1922-1927.

[96] Tueller S, Lubke G. Evaluation of structural equation mixture models Parameter estimates and correct class assignment. Struct Equ Model Multidiscip J. 2010;17:165–92. 10.1080/10705511003659318 PMC2890304 20582328

[97] Keogh RH, Daniel RM, VanderWeele TJ, Vansteelandt S. Analysis of Longitudinal Studies With Repeated Outcome Measures: Adjusting for Time-Dependent Confounding Using Conventional Methods. Am J Epidemiol. 2018;187:1085–92. 29020128 10.1093/aje/kwx311 PMC5928464

[98] Wirtz AL, Poteat T, Radix A, Althoff KN, Cannon CM, Wawrzyniak AJ, et al. American Cohort to Study HIV Acquisition Among Transgender Women in High-Risk Areas (The LITE Study): Protocol for a Multisite Prospective Cohort Study in the Eastern and Southern United States. JMIR Res Protoc. 2019;8: e14704. 31584005 10.2196/14704 PMC6802485

[99] Tillewein H, Becker J, Kruse-Diehr A. Institutional Barriers to Healthcare Services Among Transgender Individuals in the Rural Midwest. J Homosex. 2024;71:2099–115. 37289135 10.1080/00918369.2023.2222204

[100] Hubach RD, Currin JM, Giano Z, Meyers HJ, DeBoy KR, Wheeler DL, et al. Experiences of Stigma by Gay and Bisexual Men in Rural Oklahoma. Health Equity. 2019;3:231–7. 31289783 10.1089/heq.2018.0095 PMC6608693

[101] Kazyak E. Disrupting Cultural Selves: Constructing Gay and Lesbian Identities in Rural Locales. Qual Sociol. 2011;34:561–81.

[102] Sullivan PS, Khosropour CM, Luisi N, Amsden M, Coggia T, Wingood GM, et al. Bias in online recruitment and retention of racial and ethnic minority men who have sex with men. J Med Internet Res. 2011;13: e38. 21571632 10.2196/jmir.1797 PMC3221372

[103] Guest JL, Adam E, Lucas IL, Chandler CJ, Filipowicz R, Luisi N, et al. Methods for Authenticating Participants in Fully Web-Based Mobile App Trials from the iReach Project: Cross-sectional Study. JMIR MHealth UHealth. 2021;9: e28232. 34463631 10.2196/28232 PMC8441600

[104] Bauermeister J, Sullivan PS, Gravens L, Wolfe J, Countryman K, Smith-Bankhead N, et al. Reducing HIV Vulnerability Through a Multilevel Life Skills Intervention for Adolescent Men (The iREACH Project): Protocol for a Randomized Controlled Trial. JMIR Res Protoc. 2018;7: e10174. 29991470 10.2196/10174 PMC6058092

[105] MacGowan RJ, Chavez PR, Borkowf CB, Owen SM, Purcell DW, Mermin JH, et al. Effect of Internet-Distributed HIV Self-tests on HIV Diagnosis and Behavioral Outcomes in Men Who Have Sex With Men: A Randomized Clinical Trial. JAMA Intern Med. 2020;180:117–25. 31738378 10.1001/jamainternmed.2019.5222 PMC6865312

[106] Khosropour CM, Sullivan PS. Predictors of Retention in an Online Follow-up Study of Men Who Have Sex With Men. J Med Internet Res. 2011;13: e1717. 10.2196/jmir.1717 PMC3222173 21745792

[107] Zlotorzynska M, Cantu C, Rai R, Sullivan P, Sanchez T. The Annual American Men’s Internet Survey of Behaviors of Men Who Have Sex With Men in the United States. Key Indicators Report. JMIR Public Health Surveill. 2017;2020:6. 10.2196/16847 PMC7186873 32281937

[108] Zlotorzynska M, Sullivan P, Sanchez T. The Annual American Men’s Internet Survey of Behaviors of Men Who Have Sex With Men in the United States: 2016 Key Indicators Report. JMIR Public Health Surveill. 2019;5: e11313. 30785405 10.2196/11313 PMC6401665

[109] Wiatrek S, Zlotorzynska M, Rai R, Sullivan P, Sanchez T. The Annual American Men’s Internet Survey of Behaviors of Men Who Have Sex With Men in the United States: Key Indicators Report 2018. JMIR Public Health Surveill. 2021;7: e21812. 33496669 10.2196/21812 PMC7974762

[110] Anguera JA, Jordan JT, Castaneda D, Gazzaley A, Arean PA. Conducting a fully mobile and randomised clinical trial for depression: access, engagement and expense. BMJ Innov. 2016;2:14–21. 27019745 10.1136/bmjinnov-2015-000098 PMC4789688

[111] Raghunathan TE. What do we do with missing data? Some options for analysis of incomplete data. Annu Rev Public Health. 2004;25:99–117. 15015914 10.1146/annurev.publhealth.25.102802.124410

[112] Spitzer RL, Kroenke K, Williams JBW, Löwe B. A brief measure for assessing generalized anxiety disorder: the GAD-7. Arch Intern Med. 2006;166:1092–7. 16717171 10.1001/archinte.166.10.1092

[113] Weathers FW, Litz BT, Keane TM, Palmieri PA, Marx BP, Schnurr PP. The PTSD Checklist for DSM-5 (PCL-5). 2013. https://www.ptsd.va.gov/professional/assessment/adult-sr/ptsd-checklist.asp.

[114] WHO Assist Working Group. The Alcohol, Smoking and Substance Involvement Screening Test (ASSIST): development, reliability and feasibility. Addiction. 2002;97:1183–94. 12199834 10.1046/j.1360-0443.2002.00185.x

[115] Bush K, Kivlahan DR, McDonell MB, Fihn SD, Bradley KA. The AUDIT alcohol consumption questions (AUDIT-C): an effective brief screening test for problem drinking. Ambulatory Care Quality Improvement Project (ACQUIP). Alcohol Use Disorders Identification Test. Arch Intern Med. 1998;158:1789–95. 10.1001/archinte.158.16.1789 9738608

[116] Üstün TB, Kostanjsek N, Chatterji S, Rehm J. Measuring health and disability: Manual for WHO disability assessment schedule (WHODAS 2.0). World Health Organization; 2010. https://iris.who.int/handle/10665/43974. 10.2471/BLT.09.067231 PMC2971503 21076562

[117] Substance Abuse and Mental Health Services Administration. 2018 National Survey on Drug Use and Health: Methodological Summary and Definitions. Rockville, MD: Center for Behavioral Health Statistics and Quality, Substance Abuse and Mental Health Services Administration; 2019. https://www.samhsa.gov/data/sites/default/files/cbhsq-qreports/NSDUHMethodsSummDefs2018/NSDUHMethodsSummDefs2018.htm.

[118] Herek GM, Gillis JR, Cogan JC. Internalized stigma among sexual minority adults: Insights from a social psychological perspective. J Couns Psychol. 2009;56:32–43.

[119] Bockting WO, Miner MH, Swinburne Romine RE, Dolezal C, Robinson B “Bean” E, Rosser BRS, et al. The Transgender Identity Survey: A Measure of Internalized Transphobia. LGBT Health. 2020;7:15–27. 10.1089/lgbt.2018.0265 PMC6983734 31880493

[120] Fraser L, Burnell M, Salter LC, Fourkala E-O, Kalsi J, Ryan A, et al. Identifying hopelessness in population research: a validation study of two brief measures of hopelessness. BMJ Open. 2014;4: e005093. 24879829 10.1136/bmjopen-2014-005093 PMC4039863

[121] Van Orden KA, Cukrowicz KC, Witte TK, Joiner TE. Thwarted belongingness and perceived burdensomeness: construct validity and psychometric properties of the Interpersonal Needs Questionnaire. Psychol Assess. 2012;24:197–215. 21928908 10.1037/a0025358 PMC3377972

[122] Zimet GD, Dahlem NW, Zimet SG, Farley GK. The Multidimensional Scale of Perceived Social Support. J Pers Assess. 1988;52:30–41. 10.1080/00223891.1990.9674095 2280326

[123] Smith BW, Dalen J, Wiggins K, Tooley E, Christopher P, Bernard J. The brief resilience scale: assessing the ability to bounce back. Int J Behav Med. 2008;15:194–200. 18696313 10.1080/10705500802222972

[124] VanderWeele TJ. On the promotion of human flourishing. Proc Natl Acad Sci. 2017;114:8148–56. 28705870 10.1073/pnas.1702996114 PMC5547610

[125] Rosmarin DH, Krumrei EJ, Andersson G. Religion as a predictor of psychological distress in two religious communities. Cogn Behav Ther. 2009;38:54–64. 19235602 10.1080/16506070802477222

[126] Fetzer Institute/National Institute on Aging Working Group. Multidimensional measurement of religiousness/spirituality for use in health research. John E. Fetzer Institute; 1999. https://backend.fetzer.org/sites/default/files/resources/attachment/%5Bcurrent-date%3Atiny%5D/Multidimensional_Measurement_of_Religousness_Spirituality.pdf.

[127] Eden TM, Smallwood SW, Matthews DD. Using a Measurement Model to Reconceptualize the Church Experiences of Black Men who have Sex with Men. J Relig Health. 2023;62:2213–25. 36260262 10.1007/s10943-022-01671-w PMC10113399

[128] NIMH Data Archive: Prospective Cohort of Rural US LGBTQ Persons to Definitively Characterize Depression and Suicidal Ideation Burdens, Determinants, and Preferred Intervention Approaches. https://nda.nih.gov/edit_collection.html?id=4312. Accessed 7 March 2024.

